# Implementation of digital mental health interventions for children and adolescents: A systematic review

**DOI:** 10.1016/j.invent.2026.100938

**Published:** 2026-03-28

**Authors:** A. Ball, A. Rowe, K. Zieschank, S. March

**Affiliations:** a Centre for Health Research, University of Southern Queensland, Springfield, Australia; b School of Psychology and Wellbeing, University of Southern Queensland, Springfield, Australia; c Manna Institute, University of Southern Queensland, Springfield, Australia

**Keywords:** Implementation, Implementation outcomes, Implementation science, Digital mental health interventions, Children, Adolescents

## Abstract

**Background:**

Young people are adversely affected by the lack of access to mental health support. Digital interventions have demonstrated efficacy in randomised trials and pose one possible solution to increase access to mental health care. Yet, purposeful implementation efforts seldom occur, and effective interventions are rarely evaluated in real-world contexts.

**Methods:**

The Preferred Reporting Items for Systematic Reviews and Meta-Analyses guided this systematic literature review. Relevant databases were systematically searched for studies reporting on disseminated digital mental health interventions for child and adolescent anxiety and/or depression that reported implementation outcome data. Reference lists of included studies and articles citing the included studies were also searched. Intervention and implementation outcome data was extracted.

**Results:**

Nine peer-reviewed articles, pertaining to seven different digital interventions, were identified as meeting inclusion criteria. There was significant heterogeneity in methods and dissemination efforts. The use of implementation science was inconsistent across studies, and the use of implementation frameworks and models was minimal. Digital interventions were effective for and adopted by community users, though dropout rates were high. Results indicate that young people and parents find digital mental health interventions to be acceptable and beneficial.

**Conclusions:**

This review highlights the inconsistencies in the application of implementation science for scaling up digital interventions for children and adolescents. However, results of included scale-up efforts support the notion of community dissemination of child and adolescent digital interventions. Future research should focus on planning and testing implementation strategies for the dissemination of such interventions.

The review protocol was registered with PROSPERO (Registration ID CRD42024552703).

## Highlights

• Implementation research is important; yet purposeful efforts are scarce.
• Implementation Science and strategies rarely guide implementation efforts.
• Acceptability and feasibility are common collected implementation outcomes.
• Digital interventions for youth mental health are feasible for community users.

## 1 Background

For children and adolescents, access to mental health treatment is scarce internationally (Lawrence et al., 2015; McGorry et al., 2024; Mulraney et al., 2021; Pape et al., 2025). Digitally delivered interventions may increase access to care given their ability to be delivered directly to consumers through widespread dissemination (Csirmaz et al., 2024; March et al., 2018; Podina et al., 2016). Digital mental health interventions (DMHIs), with and without support, have demonstrated efficacy in reducing mental health symptoms in children and adolescents (Bennett et al., 2019; Csirmaz et al., 2024; Ebert et al., 2015; Eilert et al., 2022; Podina et al., 2016; Spence et al., 2011; Wickersham et al., 2022). Yet, despite their potential, their integration into real-world settings appears to be lacking. Implementation science can aid this process and aims to accelerate the translation of DMHIs into real-world settings by leveraging a range of theories, frameworks, and models (hereafter referred to as frameworks) to enhance the adoption, implementation, and sustainability of evidence-based practices in routine care (Graham et al., 2020; Moullin et al., 2020; Nilsen, 2015; Shelton et al., 2020).

Much of what is currently known about DMHIs is based on tightly controlled research trials that do not mimic the real world, and success in efficacy trials does not guarantee adoption of new practices (Glasgow et al., 1999). Efficacy research examines interventions in highly controlled conditions with less clinically diverse participants and often optimal user engagement (Glasgow et al., 1999). It is known that real-world users of DMHIs (i.e., children, adolescents, and their families) are complex, dynamic, and present with multi-faceted problems (March et al., 2021; Muller et al., 2022; Senyard et al., 2024) and often do not engage with DMHIs as they were intended (Neil et al., 2009; Titov et al., 2017). Interventions designed for real-world settings need to be tested in those contexts.

### 1.1 Digital Mental Health Interventions for child and adolescent mental health

DMHIs that can be delivered directly to consumers and self-directed or delivered with minimal therapist support pose a practical solution to improve child and adolescent access to mental health treatment (Muñoz et al., 2016). They also present as the easiest to scale up, given they can be delivered to large numbers of individuals and may not require integration into health services (Hermes et al., 2019; Muñoz et al., 2016). Research has indicated that such DMHIs exist and are efficacious for child and adolescent mental health (Bennett et al., 2019; Csirmaz et al., 2024; Eilert et al., 2022). Randomised trials and meta-analyses reveal that guided interventions (i.e., support from a therapist) can be equivalent to face-to-face therapy and standard treatment approaches (i.e., pharmacotherapy and psychosocial services) and are superior to waitlist and active control groups (Ebert et al., 2015; Khanna and Kendall, 2010; Podina et al., 2016; Spence et al., 2011; Wickersham et al., 2022). Symptom reduction in anxious children has also been demonstrated in self-directed (i.e., no support from a therapist) open-access interventions (March et al., 2018; Rowe et al., 2023). However, little is known about the implementation outcomes of such disseminated child and adolescent DMHIs.

### 1.2 Implementation

Scale-up and dissemination efforts can be evaluated through implementation research, which examines the effects of deliberate and purposeful actions to implement interventions (Damschroder et al., 2022; Proctor et al., 2011, Proctor et al., 2023). By collecting implementation metrics (i.e., outcomes) of scale-up efforts, researchers are better placed to understand implementation processes, success, and immediate outcomes (Proctor et al., 2011). Outcome-based frameworks, such as the Implementation Outcomes Framework (IOF; Proctor et al., 2011) and the RE-AIM framework (Glasgow et al., 1999), can be used to inform the study of the effects and outcomes of implementation efforts (Nilsen, 2015; Wilson and Kislov, 2022). The RE-AIM framework aims to understand the public health impact of an intervention through the collection of outcomes, including reach, efficacy, adoption, implementation, and maintenance. In contrast, the IOF framework focuses on understanding the processes and outcomes of implementation. Proctor et al. (2011) advanced the notion of implementation metrics by proposing a Taxonomy of Implementation Outcomes, including eight outcomes: acceptability, adoption, appropriateness, feasibility, fidelity, implementation cost, penetration, and sustainability. Proctor and colleagues' research demonstrated that implementation outcome definitions and terminology vary widely across the literature. The taxonomy aimed to provide a consensus on the use of implementation outcomes in implementation research.

Guided by Proctor et al.'s (2011) IOF, Wozney et al. (2018) conducted a systematic review that examined how the implementation of child and adolescent eMental healthcare had been studied. Their review focused on implementation trials of eHealth interventions that prevented or treated anxiety or depression, and examined any study that reported an implementation outcome. They identified 46 studies from economically developed countries that examined internet-, computer-, and smartphone-based technologies. They found that positive findings were frequently reported in relation to acceptability; however, mixed findings were reported in relation to adoption, feasibility, and fidelity outcomes. Further, acceptability, adoption, appropriateness, and feasibility of eHealth were reported to be the most frequently collected metrics. While their review provides the literature with an understanding of how and what implementation outcomes have been addressed and examined in the digital mental health literature, the majority of included studies were randomised trials. Their review did not focus on attempts to scale up and implement interventions for real-world use**.** Thus, a lack of knowledge remains regarding the implementation outcomes of real-world dissemination. A different review by Fleming et al. (2018) assessed the real-world (i.e., outside of research trials) uptake and engagement of self-guided DMHIs, but did not specifically focus on children and adolescents. They found only 10 studies that reported implementation outcomes of a publicly disseminated and available interventions, two of which focused on young people specifically. While previous reviews have examined how implementation has been studied in digital mental health care, they have focused on research trials or adult programs. The current review extends the existing research by examining scale-up efforts, with a specific focus on child and adolescent programs.

### 1.3 Objective

This review aims to examine research focusing specifically on existing efforts to scale up and disseminate DMHIs for children and adolescents into the community, and to provide an indication of the current knowledge relating to the implementation outcomes of real-world disseminated programs. By synthesising existing implementation efforts and findings, this study will inform the state of evidence relating to implementation strategies and outcomes that can be leveraged to improve the dissemination of DMHIs for child and adolescent mental health.

## 2 Methods

### 2.1 Design and Protocol Registration

The Preferred Reporting Items for Systematic Reviews and Meta-Analyses (PRISMA) statement (Page et al., 2021) was used to conduct this systematic review. A review protocol was registered with PROSPERO before commencement (Registration ID CRD42024552703).

### 2.2 Search strategy

A systematic search of the empirical literature was conducted in June 2024 for studies that examined the implementation of publicly available digital interventions for child and adolescent mental health. A title and abstract search was conducted across six electronic databases: PubMed, ScienceDirect, Web of Science, PsycINFO, PsycArticles, and Psychology and Behavioral Sciences Collection. These databases were searched from their inception, targeting literature related to mental health, digital interventions, and implementation science. Databases were searched individually using a search strategy developed by the research team in partnership with a specialist research librarian. The search strategy included terms specified by population, mental health, intervention type, intervention delivery, and implementation (see Appendix). The reference lists of included articles and articles that had cited the included articles were hand-searched for relevant literature. In April 2025, a follow-up search was run to capture any additional studies. The secondary search was conducted as the first, but was filtered by date and focused on articles published between June 2024 and April 2025.

### 2.3 Inclusion and exclusion criteria

Articles were included in the review if they met the following criteria: (1) intervention use included children and/or adolescents aged 17 years or under, or parents of children aged 17 years or under; (2) the intervention was digitally delivered, targeted anxiety and/or depression, and was disseminated in the real-world and available to community users; and (3) either had as a specific aim to test or report on implementation of digital intervention (e.g. using implementation framework), or, reported data on implementation metrics, including outcomes (i.e., acceptability, adoption, feasibility, effectiveness data etc.). Articles were excluded if: (1) the intervention was designed for use only by people 18 years or over (unless intended as a parent program); (2) the intervention was a population-level prevention program where all users received the intervention regardless of symptomology (e.g., school-based trials that deliver the intervention as part of the school curriculum); (3) the intervention was not digital, did not target depression or anxiety, or was not community disseminated (i.e., not available to the public/community), or if the intervention was not available to users outside of a tightly controlled research trial; (4) did not report data on any aspect of implementation including effectiveness; and (5) non-English papers, abstract only papers, conference proceedings, systematic reviews, or study protocols.

Implementation research, and determining real-world efficacy, is encouraged only after having first established the efficacy of an intervention (Lane-Fall et al., 2019). Thus, inclusion criteria narrowed the focus of this review to include only interventions that targeted anxiety and depression alone given the efficacy of such interventions is well established in the literature (Bennett et al., 2019; Csirmaz et al., 2024; Eilert et al., 2022; Fernández-Batanero et al., 2025). It is recognised that mental health encompasses a broad range of issues, and many DMHI's exist for a range of mental health concerns, yet the established literature focuses heavily on digital interventions that target anxiety and depression (Hollis et al., 2017).

### 2.4 Study selection

The first author (AB) and a third-year practicum student (GT) independently screened titles and abstracts of all retrieved articles. Articles were included for full-text review if both authors identified them as potentially relevant. Author SM guided the screening process. Discrepancies were resolved via discussion with authors AR and KZ, who were independent of the screening process. In the second phase of screening (full-text review), all articles were reviewed by at least two authors from AB, AR, and KZ. Reasons for exclusion were recorded during full-text review.

### 2.5 Data extraction and synthesis

A data extraction spreadsheet was developed and used, where data was extracted by the first author (AB) and cross-checked by authors SM, AR, and KZ. Discrepancies were resolved by discussion. The extracted data included article reference details, study and intervention characteristics, implementation outcomes, implementation strategies (when used), and study results. Due to the heterogeneity of outcomes measured and the measurement tools used across the included articles, a meta-analysis was deemed inappropriate. Therefore, to consolidate the findings, a narrative synthesis was conducted, synthesising the outcomes outlined by Proctor et al. (2011).

### 2.6 Quality appraisal

The Mixed Methods Appraisal Tool (MMAT; Hong et al., 2018) was deemed appropriate for study appraisal, given the diversity of research designs among the included studies. The MMAT is a valid and reliable tool for mixed-method reviews, focusing on evaluating key methodological criteria (e.g., sample, sampling methods, and appropriate measurement) to critically assess methodological quality (Hong et al., 2019). The updated version (Hong et al., 2018) was used, which employed two screening questions and five unique criteria to assess the quality of each study. The MMAT is used to evaluate five different research designs; the included studies were evaluated according to their methodological domain. Each study was independently assessed against the MMAT by authors AB and AR. Methodological quality was presented as a percentage.

## 3 Results

### 3.1 Study selection

**Fig. 1:**
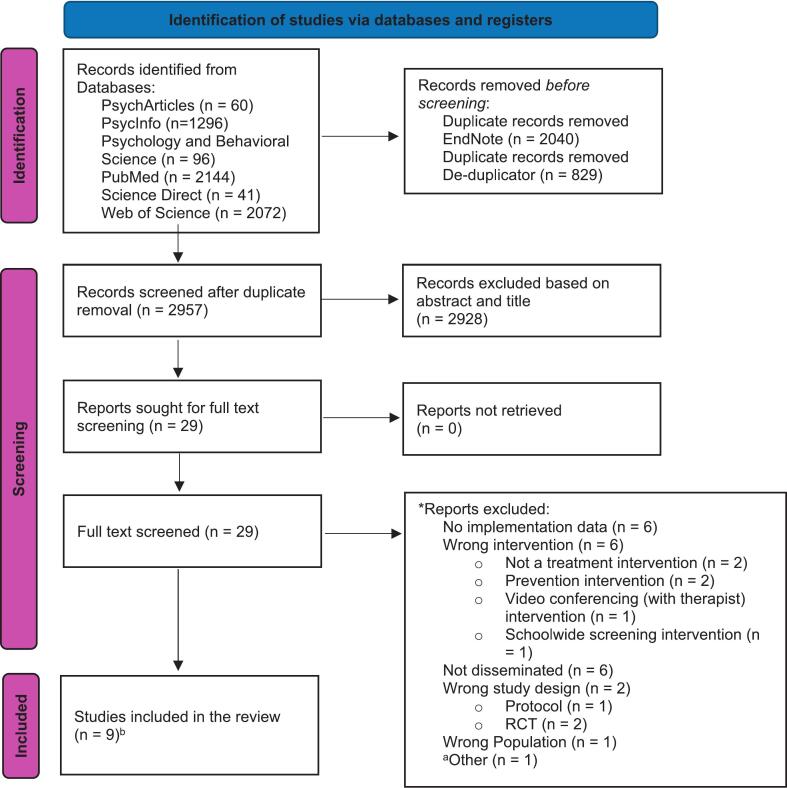
PRISMA flow diagram. *Note*. *Papers had multiple reasons for exclusion; only the main reason was reported. ^a^Thesis, relevant study included as published study, no requirement for entire thesis/other studies within. ^b^Final sample (*n* = 9) included the additional study found from articles that had cited the included studies; and the two additional from the 2025 search re-run.

Results of the search and screening process are detailed in Fig. 1. The database search yielded 5826 articles, of which 2957 titles and abstracts were screened after duplicates were removed. Title and abstract screening excluded 2928 articles; 29 full texts were sought for retrieval and were reviewed. Six articles met the inclusion criteria and were included in the review. The 2025 follow-up search yielded 999 additional studies, of which 600 titles and abstracts were screened after duplicates were removed. Title and abstract screening excluded 597 articles; three full texts were reviewed, of which two were included. One study by Alvarez-Jimenez et al. (2024) clearly stated, ‘the implementation strategy and outcomes will be reported elsewhere’, though the paper did report the engagement, clinical outcomes, and satisfaction of a real-world dissemination effort. Although the explicit implementation aspect of their evaluation was not reported in the paper, it provided data suitable for this review and was included. The Eisen et al. (2013) paper formed part of a randomised controlled trial (RCT); however, it was included in this review because it was not a tightly controlled trial where the setting or user was not representative of real-world use; all participants, regardless of condition, received the same digital treatment (CATCH-IT), and this paper in particular aimed to explore the implementation effort.

An article by Spence et al. (2019) discussed the BRAVE Self-Help program and was publicly disseminated; however, it was not included because it was a secondary analysis of data captured by the March et al. (2018) study, which was already included in the review. Neil et al. (2009) studied predictors of adherence and compared module and exercise completion in two different adolescent samples. While one sample was a community sample, this paper was not an evaluation of an implementation or scale-up effort and did not report effectiveness or other implementation metrics in the community; thus, it was excluded. While MoodGYM is a publicly available digital intervention available to young people, to our knowledge, there are no studies that address the implementation of the program when disseminated in the community (i.e., evaluation outside of randomised controlled trials or school-based prevention trials). The reference lists of included articles were screened; however, no articles were identified from this search. Articles that had cited the included articles were searched for inclusion; one additional study was found and included (March et al., 2021).

### 3.2 Quality appraisal

Quality appraisal results are presented in Table 1. The included studies fit within the quantitative descriptive methodological domain of the MMAT. Studies were within a high-quality range (≥80%) according to the MMAT criteria. Descriptive studies were most frequently assessed as having either minimal or no information pertaining to the risk of nonresponse bias. Community users of DMHIs frequently discontinue treatment after a single session (Batterham et al., 2008). Consequently, it is anticipated that studies would exhibit high nonresponse rates. However, only three descriptive studies provided a clear explanation for handling missing data.

**Table 1:** MMAT quality appraisal criteria.

Author, year	Quantitative descriptive studies					Quality score
	4.1	4.2	4.3	4.4	4.5	
Moor et al. (2019)	Yes	Yes	Yes	Yes	Yes	100%
Vigerland et al. (2024)	Yes	Yes	Yes	Yes	Yes	100%
March et al. (2018)	Yes	Yes	Yes	Yes	Yes	100%
Schleider et al. (2020a)	Yes	Yes	Yes	No	Yes	80%
Shroff et al. (2023)	Yes	Yes	Yes	No	Yes	80%
Eisen et al. (2013)	Yes	Yes	Yes	Can't tell	Yes	80%
March et al. (2021)	Yes	Yes	Yes	Can't tell	Yes	80%
Gee et al. (2024)	Yes	Yes	Yes	No	Yes	80%
Alvarez-Jimenez et al. (2024)	Yes	Yes	Yes	No	Yes	80%

*Note*. 4.1 = is the sampling strategy relevant to address the research question?; 4.2 Is the sample representative of the target population?; 4.3 = Are the measurements appropriate?; 4.4 = Is the risk of nonresponse bias low?; 4.5 = Is the statistical analysis appropriate to answer the research question?

### 3.3 Study and intervention characteristics

#### 3.3.1 Study characteristics

Characteristics of each included study are presented in Table 2. This review identified nine articles related to seven disseminated digital interventions, highlighting the scarcity of implementation research in the child and adolescent DMHI literature. A total of 33,362 people participated in the nine studies; 63 of these participants were providers of the intervention (Eisen et al., 2013), 23,177 children and adolescents engaged with at least one session of an intervention, and of the total engaged participants, 10,366 were drawn from one study (March et al., 2021). The included studies occurred in economically developed Westernised countries, including the USA (*n* = 3), Australia (*n* = 3), New Zealand (*n* = 1), the United Kingdom (*n* = 1), and Sweden (*n* = 1). Participants ranged in age from 7 to 25 years, with two studies and interventions including users over the age of 18 years (Alvarez-Jimenez et al., 2024; Moderated Online Social Therapy [MOST]; Eisen et al., 2013; CATCH-IT). Studies were conducted as open-access disseminations (*n* = 4) or in primary health care settings (*n* = 5). Implementation was most often examined using an open trial or naturalistic evaluation (*n* = 8).

**Table 2:** Characteristics of each included study.

Publication	Intervention and eligibility criteria	Study Design, aims, and measures	Sample
Alvarez-Jimenez et al., 2024	• MOST • Professional referral from youth mental health service clinician or administrative staff. No eligibility criteria for referral	• Observational Real-world Evaluation • Aim: Real-world evaluation of the first 32 months of MOST implementation. Measures • PHQ-4 • K-10 • SWEMWBS • Satisfaction Questionnaire	*N* = 13,792 (engaged sample = 5709) of Australian general population • Age range = 12–25 years • Mean age = 17.5 years • 64% Female
Eisen et al., 2013	• CATCH-IT • Access through primary care screening with positive screen for subthreshold depression symptoms (at least 1 core symptom of depressive disorder for at least 2 weeks)	• Randomised Clinical Trial • Aim: explore the implementation & public health impact of CATCH-IT when delivered in primary care using the RE-AIM Framework Measures • CES-D • Provider Knowledge and Attitudes Survey	*N* = 146 • Providers = 63 (36 Nurses/Medical assistants, 27 Primary Care Physicians) • Adolescents = 83 of United States of America (USA) Midwest and Southeast general population • Age range = 14–21 years mean age 17.5 years • 56.2% female
Gee et al., 2024	• Lumi Nova • Access through referral from UK National Health Services • Engaged during an 18-month period July ‘21 & December ‘22, aged 7–12 years, use the intervention for at least one week, residing in the UK	• Observational Real-world Evaluation • Aim: better understand how Lumi Nova has been used in real-world settings. Measures • CORS	*N* = 1029 (engaged sample = 644) of United Kingdom general population • Age range = 7–12 years • Mean (*SD*) age = 9.71 (1.46) years • 51.79% female • 48.21% male • 79 missing
March et al., 2018	• BRAVE Self-Help • Elevated anxiety as determined by scores on CAS-8 (≥84th percentile or T-score greater than ≥60). No professional referral required	• Exploratory Observational study • Aim: feasibility and acceptability of BRAVE Self-Help when widely disseminated & self-sought by users Measures • CAS-8 • Satisfaction questionnaire (author-developed)	*N* = 4425 of Australian general population • Age range = 7–17 years • Mean (*SD*) age 12.95 (2.91) years • 7–12 years (*n* = 1473) • 13–17 years (*n* = 2952) • 66.39% Female • 31.77% Male • 1.84% Other
March et al., 2021	• BRAVE Self-Help • Elevated anxiety as determined by scores on CAS-8 (≥84th percentile or T-score greater than ≥60). No professional referral required.	• Single-cohort longitudinal open trial • Aim: determine changes in anxiety through examination of symptom trajectory in users of BRAVE Self-Help when widely disseminated and self-sought Measures • CAS-8	*N* = 10,366 of Australian general population • Mean (*SD*) baseline CAS-8 score 15.11 (3.36) Child program (7–12 years) • *n* = 4140 • Mean (*SD*) age 9.34 (1.48) • 52.95% male, 47.05% female Adolescent program (12–17 years) • *n* = 6226 • Mean (*SD*) age 14.55 (1.66) • 23.77% male, 72.79% female, 3.44% other
Moor et al., 2019	• BRAVE_Thearpist Assisted (TA) • Non-elevated and elevated anxiety according to Australian norms on the SCAS. Professional referral required (family doctor, school nurse, child mental health service).	• Open effectiveness trial • Aim: uptake and feasibility of BRAVE_TA in primary care in Canterbury, New Zealand (NZ) Measures • CAS-8 (Parent and Child)	*N* = 1026 of Canterbury NZ general population • Age range = 7–17 years • Mean (*SD*) = 12.2 (3.1)
Schleider et al., 2020a	• Project YES • No eligibility criteria besides age. No professional referral required.	• Nonrandomised exploratory program evaluation • Aim: evaluate Project YES when widely disseminated and self-sought Measures • SMFQ • State Hope Scale • Beck Hopelessness Scale-4 • Self-Hate Scale • Perceived Control (Author-developed single-item measure) • Program Feedback Scale	*N* = 694 of USA general population • Age range = 10–17 years • 542 females, 142 males, 2 intersex, 9 missing information • <13 years = 99 • 14 to <18 years = 594 • 1 missing information
Shroff et al., 2023	• Project YES • Reside in the San Antonio and Texas areas. No professional referral required.	• Nonrandomised exploratory program evaluation • Aim: evaluate culturally adapted version of Project YES when disseminated in community • Outcome Measures – Mood and Feelings Questionnaire-Short – State Hope Scale – Beck Hopelessness Scale-4 – Self-hate Scale – Program Feedback Scale	*N* = 1801of San Antonio, Texas general population • Age range = 11–17 years • English version: 1705 (53.96% female) • Spanish version = 96 (43% female)
Vigerland et al., 2024	• BIP Anxiety and BIP OCD • Access through health service clinical interview to assess diagnosis – Inclusion: meet diagnostic criteria for any anxiety disorder. – Exclusion: severe depression, high risk of suicide, and/or psychosocial problems like substance abuse or maltreatment	• Open trial with naturalistic evaluation • Aim: evaluate BIP Anxiety and BIP OCD in child and adolescent health services in Region Jämtland Härjedalen, Sweden Measures • CGI-S • CGI-I • CGAS • RCADS-C/P • WSAS-Y/P	*N* = 83 of Region Jämtland Härjedalen, Sweden general population • Age range = 8–17 years • Mean (*SD*) age = 13.43 (2.51) • 64 females, 19 males

*Note*. PHQ-4 (Patient Health Questionnaire-4), SWEMWBS (Short Warwick-Edinburgh Mental Wellbeing Scale), K-10 (Kessler Psychological Distress Scale-10), CES-D (Centre for Epidemiological Studies Depression), CORS (Child Outcome Rating Scale), CAS-8 (Child Anxiety Scale), SMFQ (Short Mood and Feelings Questionnaire), PHQ-2 (Patient Health Questionnaire-2), CDI (Children's Depression Inventory), CGI-S (Clinical Global Impressions — Severity), CGI-I (Clinical Global Impressions — Improvement), CGAS (Children's Global Assessment Scale), RCADS-C/P (Revised Children's Anxiety and Depression Scale — Child and Parent version), WSAS-YP (Work and Social Adjustment Scale — youth and parent version).

#### 3.3.2 Intervention characteristics

Intervention characteristics are presented in Table 3. Seven interventions were identified: Four targeted anxiety (BRAVE Therapist-Assisted, BRAVE Self-help, Lumi Nova, & BiP Anxiety), two targeted depression (Project YES & CATCH-IT), and one targeted anxiety and depression (MOST). One intervention drew from Social Psychology (Project YES; see Schleider et al., 2020b); however, CBT was the most common therapeutic modality (6/7 interventions). CBT-based interventions included psychoeducation, anxiety management techniques, exposure strategies, behaviour and thought monitoring, and relapse prevention.

**Table 3:** Intervention characteristics.

Publication	Intervention	Target concern	Target population	Therapeutic modality	Therapist involvement	Parent involvement	Delivery mode	Intervention length	Navigation	Accessibility
Alvarez-Jimenez et al., 2024	MOST	Anxiety & depression	12 to 25 years	CBT	Yes ≤14 years old | By choice ≥15 years old	Yes ≤14 years old | Nil ≥15 years old	Web-based	1 of 6 journeys (length of not clear)	Open navigation	Free access through professional referral
Eisen et al., 2013	CATCH-IT	Depression	14 to 21 years	Behavioural activation, CBT, interpersonal psychotherapy, & community resiliency concept model	Provider brief advice OR motivational interview	Yes	Web-based	14 modules	Sequential	Access through primary care setting (costs not clear)
Gee et al., 2024	Lumi Nova	Anxiety	7 to 12 years	CBT	Nil	Yes	App-based	3 goals to work through	Open navigation	Free access through practitioner referral
March et al., 2018	BRAVE self-help	Anxiety	7 to 17 years	CBT	Nil	Yes	Web-Based	Child/teen: 10 modules Parent (teen): 5 modules Parent (child): 6 modules	Sequential	Free access through self or professional referral
March et al., 2021	BRAVE self-help	Anxiety	7 to 17 years	CBT	Nil	Yes	Web-based	10 modules + 2 booster sessions	Sequential	Free access through self or professional referral
Moor et al., 2019	BRAVE therapist assisted	Anxiety	7 to 17 years	CBT	Yes	Yes	Web-based	Child/teen: 10 modules Parent (teen): 5 modules Parent (child): 6 modules	Sequential	Free access through professional referral
Schleider et al., 2020b	Project YES	Depression (sad mood and low self-esteem)	11 to 17 years	See Schleider et al., 2020b	Nil	Nil	Web-based	1 module (30 min)	–	Free access through self-referral
Shroff et al., 2023	Project YES	Depression (sad mood and low self-esteem)	11 to 17 years	See Schleider et al., 2020b	Nil	Nil	Web-based	1 module (30 min)	–	Free access through self-referral
Vigerland et al., 2024	BIP anxiety and BIP OCD	Anxiety	8 to 17 years	CBT	Yes	Yes	Web-based	12 modules each (parent and child)	Sequential	Access through primary care setting (costs not clear)

Interventions were web- and app-based and delivered entirely digitally. Four interventions delivered sequential modules (ranging from 10 to 12 sessions), intended to be completed weekly. All interventions were designed to be engaging and included text, images, videos, and interactive materials. Project YES was delivered as a single session intervention (SSI), where users chose one of three 30-min sessions (i.e., The ABC Project, Project Personality, Project CARE). Both MOST and Lumi Nova were delivered as open-navigation interventions.

Parental involvement was encouraged in four interventions through the delivery of parental modules (i.e., BRAVE Therapist-Assisted and BRAVE Self Help & BiP Anxiety) or parental workbooks (i.e., CATCH-IT). Parents were required to activate the Lumi Nova intervention, but young people were encouraged to complete the intervention on their own. Three interventions included therapist support (MOST, Alvarez-Jimenez et al., 2024; BRAVE Therapist-Assisted, Moor et al., 2019; BiP Anxiety, Vigerland et al., 2024), while the remaining four were delivered in a self-directed format. Support ranged from homework review, feedback, and encouragement (BiP Anxiety & BRAVE Therapist-Assisted) to hands-on support that involved direct therapist contact (BiP Anxiety & MOST). Majority of interventions required a professional referral, with only two allowing for self-referral into the intervention (BRAVE Self-Help [March et al., 2018; March et al., 2021] & Project YES [Schleider et al., 2020b; Shroff et al., 2023]).

### 3.4 Implementation outcomes addressed by studies

**Fig. 2:**
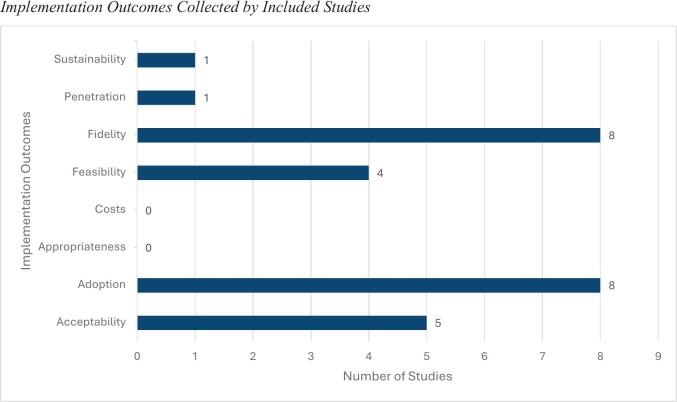
Implementation outcomes collected by included studies.

To identify the current state of implementation within the child and adolescent DMHI field and the outcomes being reported, an outcome-based framework was used to determine the outcomes in the included studies. Fig. 2 is mapped to Proctor et al.'s (2011) implementation outcomes and presents the implementation outcomes collected in each study. No study explicitly referred to Proctor's model as a guide to data collection, though this framework captured the outcomes collected in the studies. No study reported all eight outcomes; however, all but two implementation metrics (i.e., costs and appropriateness) were collected by at least one study. Fidelity (8/9 studies; 89%), adoption (8/9; 89%), and acceptability (5/9; 55%) were the most frequently reported outcomes; penetration and sustainability were reported in one study (Eisen et al., 2013). Four of the nine studies collected data pertaining to providers (i.e., intervention providers or refers) and end users (i.e., children and adolescents and/or parents; Eisen et al., 2013; March et al., 2018; March et al., 2021; Moor et al., 2019), and the remaining studies reported on end users only (Alvarez-Jimenez et al., 2024; Gee et al., 2024; Schleider et al., 2020a; Shroff et al., 2023; Vigerland et al., 2024). The implementation outcomes of each study are summarised in Table 4 for each intervention and synthesised below.

**Table 4:** Implementation metrics, outcomes, and limitations of each included study.

Publication	Implementation metrics	Findings	Limitations
Alvarez-Jimenez et al., 2024	• Adherence: Logins • Program Satisfaction: 9-item rating scale • Clinical Effectiveness: clinical change from pre-to-post intervention (12 weeks).	Effectiveness: • 2648 completes • Mean (*SD*) baseline PHQ4 score = 7.75 (2.85) • Mean (*SD*) 12-week PHQ4 score = 6.46 (3.13) • Significant reduction of depression and anxiety on PHQ4 (Cohen's *d* = 0.41) • Significant clinical improvement = 44% of users Adherence: • Mean 12-week session count (logins) = 19.26 (*SD* = 41.34) • at least 14 days of use = 71.7% of users • at least 12 weeks of use = 40.1% of users • at least 24 weeks of use = 18.8% of users • Viewing of psychotherapeutic content = 65% of users • Messages to clinicians = 38% of users Satisfaction: • Mean score > 3.5 (out of 5) on 7 of 8 satisfaction domains (*n* = 1495)	• Depression and anxiety measured and analysed together
Eisen et al., 2013	• RE-AIM Framework – Adoption: proportion of physicians approached who participated – Reach: proportion of at-risk adolescents identified by screening process – Efficacy: mean effect size ^b^ of CES-D change at 3 months – Implementation: proportion of adolescents completing intervention – Maintenance: proportion of primary care sites that agreed to continue intervention • Public Health Impact – RE-AIM factors multiplied to calculate the public health impact • Previous Knowledge and Provider Attitudes – Knowledge = true/false response – Provider attitudes = 5-Point Likert Scale	Provider knowledge and attitudes: • Knowledge of the intervention: mean percent correct – Nurses = 39.15% – Physicians = 63.16% • Provider attitudes mean response = neutral/agree range • Willingness ratings = strongly disagree/disagree range • Capability ratings = Nurses significantly higher ratings than physicians (*p* = .02) RE-AIM Statistics • Adoption = 0.50 to 1.00 • Reach = 0.30 to 1.00 • Efficacy = 0.19 to 1.86 • Implementation = 0.00 to 1.00 • Maintenance = 85% of all sites agreed to continue the intervention Public Health Impact = 0.00 to 0.59 Mean baseline CESD-10 score = 24.4 (*SD* = 12.6)	• Minimal information drawn from child and adolescent users • Effect size used to describe effects was not explicit • Did not report post-intervention CES-D mean scores • Reach = number of people screened for risk of depression not number of people who engaged with treatment program • Not explicit on where the RE-AIM statistics were derived from
Gee et al., 2024	• Adherence: number of sessions & challenges completed • Uptake: referral type, engagement • Effectiveness: change in anxiety from pre-intervention to last measurement data point	Effectiveness: • *n* = 123 completes • Statistically significant increase in CORS scores (Cohen *d* = 0.46) • Reliable improvement = 37.34% • Reliable deterioration = 9.8% • No reliable change = 52.8% Uptake: • 1029 people referred • 644 activated game key • Self-sign up = 381 • Clinician referral = 263 Adherence: • Median number of sessions = 6 (IQR 3–12) • Median total time spent = 41 (IQR 15–79) minutes • Median number of challenges = 1 (IQR 0–3) • Completed at least one session = 58.1%	• Did not report pre-or-post-intervention CORS mean scores • No explicit mention of an implementation strategy or framework used • Limited implementation outcomes collected
March et al., 2018	• Program satisfaction and acceptability: author developed 5-item rating scale • Adherence: the number of sessions completed • Referral patterns: how the user came to the program • Effectiveness: change in anxiety score across time = mean change in raw scores, reliable change index, & proportion of youth crossing clinical threshold	Effectiveness: • Mean (*SD*) CAS-8 score – Baseline: child = 13.92 (2.96) & adolescent = 15.66 (3.30) – Final: child = 10.94 (4.82) & adolescent = 12.88 (5.05) • Statistically significant average decrease of 3 points on the CAS-8 for both child (Cohen *d* = 0.66) & adolescent (Cohen *d* = 0.65) • Elevated range to non-elevated post-treatment = 53.88% • Clinical range to nonclinical post treatment = 59.1% Program Satisfaction: • Mean (*SD*) total satisfaction rating = 17.72 (5.16) Adherence: • 24.75% of participants with elevated anxiety at baseline provided data at session 4 (2nd time point) • 21% of registered users did not go on to complete the first session • 48.05% completed 1–2 sessions • 30.31% completed ≥3 sessions. Referral patterns: • School-based professional = 28.67% • Word of mouth, radio, magazine, or advertisements = 28.70% • External health professional =13.36% (GP =13.96%, psychologist = 55.67%, & social worker = 9.60%) • Parent or family member = 10.85% • Internet searching = 9.94% • Beyondblue = 8.48%	• No explicit mention of an implementation strategy or framework used • Limited implementation outcomes collected
March et al., 2021	• Program Adherence: the number of completed sessions • Referral patterns: how the user came to the program • Effectiveness: reductions in anxiety symptoms across program	Effectiveness: • Mean (*SD*) baseline CAS-8 score – child = 14.16 (3.04) – adolescent = 15.75 (3.42) • HAS: Intercept = 19.72 (SE 0.09) estimate = −1.39; SE 0.11; *P* < .001 • MAS: Intercept = 13.75 (SE 0.04), with a negative slope (estimate = −0.97; SE 0.04; *P* < .001) Program adherence: • Adherence decreased with program progression • Completed 2 sessions = 50% Referral patterns: • School-based professionals = 33.57% • External health professionals = 19.03% • Parents, friends, or family = 12.83% • Beyondblue = 6.22% • Self-referred through internet searching = 8.1% • Word of mouth, radio, magazine, or advertising = 6.52%	• Limited implementation outcomes reported • No explicit mention of an implementation strategy or framework used
Moor et al., 2019	• Uptake – Referral patterns: source of referral, monthly referral numbers, and appropriateness of referral based on level of anxiety – Non-engagement: No. of referred people who did not start or did not complete one session • Program Progression: No. of sessions completed • Feasibility: referral patterns, non-engagement rates, program progression • Effectiveness: extent of change from baseline to last completed CAS-8 (only users with paired data were included in analysis)	Effectiveness: • *n* = 438 completes • Baseline anxiety in mild to elevated range • Effect sizes: • <11 years – Males: Cohen *d* = 0.83 – Females Cohen *d* = 0.62 • >11 years – Males: *d* = 0.58 – Females: *d* = 0.64 • Completed ≥9 session (*n* = 69) – 84% = decreased anxiety score – 16% = same or worse anxiety score Feasibility: • Steady referral rates (≈ 30 referrals/month) Referral patterns: • Total = 1361 • Ineligible = 48 • Family doctors = 65% of referrals • Public health nurse in schools = 19% of referrals • Community-based child health NGO = 16 Non-engagement: • Referrals contacted = 1261 • Referrals not engaged = 235 Program progression • Mean no. of completed sessions = 4.4 • <1 session = 163 users • ≥1 session = 863 users – 54% = 4 sessions – 24% = 7 sessions – 17% = 9 or more sessions	• No explicit mention of an implementation strategy or framework used • No information reported from the perspective of providers • Reach limited due to specific locality of dissemination • Effectiveness analysis looked only at users with paired data, with no inclusion of an intent-to-treat analysis
Schleider et al., 2020b	• Effectiveness: change in the SSI outcomes from pre-SSI to post-SSI • Acceptability and feasibility: determined by Program Feedback Scale. Mean score > 3 across all items = SSI acceptability • Usage patterns: – No. of users who began Project YE, selected, started, completed an SSI, & which SSI • Feasibility: overall & item level means for each SSI using completion data and outcomes of PFS	Effectiveness • *n* = 187 completer sample – Mean depression score = 16.51 (*SD* = 6.34; clinically elevated according to SMFQ cut-off score of ≥11) • Across all SSIs significant pre-to-post SSI improvements: Cohen *d*_av_ & *d*_z_ – Agency = *d*_av_ = 0.39; *d*_z_ = 0.50 – Hopelessness = *d*_av_ 0.53; *d*_z_ = 0.71 – Self-hate = *d*_av_ = *0*.32; *d*_z_ = 0.61 – perceived control = *d*_av_ = 0.60; *d*_z_ = 0.72 Usage patterns: • Overall completion rate of 34.32% • Registered = 694 • Selected SSI = 612 Began SSI = 539 • Finished SSI = 187 Acceptability: • All item level means >3.50/5 = acceptable	• No explicit mention of an implementation strategy or framework used • Assessment responses collected approximately 30 min apart. No extended follow-up points • No program feedback from users who dropped out or did not go on to start an SSI. • Proportion of sample who were within clinical range of depression was not reported
Shroff et al., 2023	• Effectiveness: Change in pre-to-post-SSI levels of agency, hopelessness, & self-hate • Use patterns: No. of children/adolescents who started, chose, began, and completed an SSI • Acceptability and feasibility: Determined by Program Feedback Scale. Mean score > 3 across all items = SSI acceptability	Effectiveness: English version *n* = 797: • Pre-SSI Means (*SD*s) – agency = 5.3 (1.7) – hopelessness = 1.0 (0.9) Self-hate = 2.5 (1.7) • Post-SSI Mean (*SD*s) – agency = 5.7 (2.0) – hopelessness = 0.8 (0.9) – self-hate = 2.1 (1.6) • Significant improvements in all pre-to-post SSI outcomes: small-medium effects (Cohen *d*_*z*_*)* – Agency *=* 0.2 – Hopelessness = 0.33 – Self-hate = 0.27 Spanish version *n* = 35: • Pre-SSI Means (*SD*s): – agency = 5.8 (1.8) – hopelessness = 1.0 (0.8) – self-hate = 2.1 (1.5) • Post-SSI Means (*SD*s) – Agency = 5.9 (2.1) – Hopelessness = 0.9 (1.0) – Self-hate = 1.8 (1.6) • Significant improvements in self-hate pre-to-post-SSI: small-medium effect (Cohen *d*_z_ = 0.37). No significant improvements in agency or hopelessness. Use patterns: • English version: – Registered = 1705 – Began SSI = 1464 – Completed SSI = 855 • Spanish version: – Registered = 96 – Began SSI = 77 – Completed SSI = 39 Acceptability: • English version: – *n* = 794 (SSI & PFS completer sample), mean (*SD*) of the PFS = 3.84 (0.88). • Spanish version: – *n* = 37 (SSI & PFS completer sample), mean (*SD*) of the PFS = 4.15 (0.83).	• No explicit mention of an implementation strategy or framework used • Assessment responses collected approximately 30 min apart. No extended follow-up points • No explicit indication as to whether changes were clinically meaningful • No program feedback from users who dropped out or did not go onto start an SSI.
Vigerland et al., 2024	• Acceptability: proportion of people who accepted the offer of iCBT & proportion of users who dropped out • Treatment satisfaction: post-treatment questionnaire • Adherence: no. of completed modules & the clinical rated questionnaire Internet Intervention Patient Adherence Scale • Effectiveness: change from baseline to three-month follow-up • Feasibility: – Clinician time per participant – Additional therapist support during treatment phase – adverse events	Effectiveness • Significant improvement from baseline to follow-up on all measures. • Means (*SD*s) for CGI-S – Baseline = 4.80 (0.75), *n* = 83 – Post-intervention = 3.72 (0.13), *n* = 68 – Follow-up = 2.97 (1.53), *n* = 63 • Estimated change on CGI-S from baseline to follow up = −1.84 (*p* < .001, Cohen *d =* 2.45) • 34% = at least ‘much’ improvement post treatment • 40% = at least ‘much’ improvement at three-month follow-up • 25% = remitters at post-treatment • 43% = remitters at three-month follow-up • Three-month follow-up: – Patients discharged from clinic = 37% – Further support for anxiety or other disorders = 13% Acceptability: • Dropped out: 14 people Satisfaction: • 89% of young people (*N* = 47) and 97% of parents (*N* = 58) = ‘at least ok’ Adherence: • Average number of completed sessions: – Young people = 7.5/12 – Parents = 8/12 • Completed ≥4 modules: – Young people = 67 – Parents = 70 • Completed 12/12 modules: – Young people = 10 – Parents = 17 Feasibility: • Clinician time: Minutes per week – Children: Mean (*SD*) = 13.03 (13.48) – Parents: Mean (*SD*) = 15.2 (13.7) • Additional support: 35% of participants • Adverse events: – ≥1 adverse event: 33% of children – ≥1 adverse event: 27% of children	• Small sample • Disseminated in a rural location with small population density (generalisability to other care services limited)

### 3.5 Sustainability

Sustainability refers to the extent to which an implemented intervention is continued or maintained in the setting it was implemented (Proctor et al., 2011). For example, whether the intervention was continued after the initial implementation period. Maintenance data was collected in Eisen et al.'s (2013) study, the only study to explicitly refer to the use of an implementation framework in their evaluation. Their study was guided by the RE-AIM framework (Glasgow et al., 1999), which emphasises the reporting of ‘maintenance data’, which directly maps to Proctor et al.'s (2011) ‘sustainability’ outcome. Findings of this study show that 85% of all sites involved agreed to continue the program after the implementation period. No other study reported on sustainability.

### 3.6 Penetration

Penetration is defined as an intervention's integration within the context in which it is implemented (Proctor et al., 2011) and is often captured by reach data. For example, the number of users reached within a target population. Reach data was collected in Eisen et al.'s (2013) study, however, it is essential to highlight, that reach (i.e., penetration) was only measured as the proportion of adolescents who were identified via screening as being at risk for depression rather than the proportion of people who were reached by the treatment program (not all who were identified went on to do the treatment component). While other studies were not explicit in their intent to collect penetration data, from a reach perspective, all included studies reported data that indicated who the platform was reaching (i.e., user numbers & characteristics), which may also represent an intervention's penetration within the community. Findings across studies suggest DMHIs reach a wide array of end-users (i.e., children, adolescents, young adults), parents, and providers (i.e., school staff, family doctors, therapists, or other health practitioners). Eligibility criteria differed across studies; however, all focused on young people with experiences of anxiety or depression. On average, users reached were within an elevated to clinically elevated range of anxiety or depression pre-intervention in seven of the nine studies, though only three studies required elevated levels of anxiety to engage with treatment content (March et al., 2018; March et al., 2021; Vigerland et al., 2024). One study required users to demonstrate only subthreshold depression symptoms (i.e., no major depression (Eisen et al., 2013); on average, users in this study were at risk for clinical depression. Gee et al. (2024) did not report pre-intervention scores; therefore, it was unclear whether users were within a clinical range prior to the intervention. Overall, the sample sizes within the studies indicate varying levels of platform penetration, ranging from 80 to over 10,000 users spread across both urban and rural locations.

### 3.7 Fidelity

Fidelity refers to the intervention having been implemented as intended (Proctor et al., 2011), for example, whether participants completed the intervention modules as prescribed. Fidelity was commonly collected, with eight out of nine (88.8%) studies reporting on this outcome; however, the studies reported in this review did not explicitly utilise the term fidelity; instead, studies collected and referred to patient adherence (Alvarez-Jimenez et al., 2024; Gee et al., 2024; March et al., 2018; March et al., 2021; Vigerland et al., 2024), program progression (Moor et al., 2019), and completion rate (Schleider et al., 2020a; Shroff et al., 2023) data. The findings revealed that for the interventions with multiple sessions, adherence decreased with program progression (March et al., 2018; March et al., 2021; Moor et al., 2019; Vigerland et al., 2024). In many of the studies, approximately half of the users proceeded to complete two or more sessions. Across the studies and interventions, typically less than 30% of the intervention was completed by users. In the SSI studies, more than half of the registered users did not go on to complete the single session (Schleider et al., 2020a; Shroff et al., 2023). Some studies have revealed that, on average, users who completed more sessions tend to be younger and exhibit lower baseline anxiety than users who completed fewer sessions (March et al., 2018; March et al., 2021; Moor et al., 2019). Similarly, Schleider et al. (2020a) reported that users who went on to complete the single session were on average younger. On average, adherence was lower in self-guided interventions with multiple sessions (March et al., 2018; March et al., 2021) compared to supported interventions (Moor et al., 2019; Vigerland et al., 2024). No clear patterns of differential adherence/fidelity were identifiable across mental health symptomology due to heterogeneity in intervention delivery (i.e., single session vs multiple session vs open navigation).

### 3.8 Feasibility

Feasibility is the degree to which an intervention can be or is successfully delivered or used (Proctor et al., 2011). For example, whether the setting in which an intervention is delivered was suited to the intervention. Feasibility may include measures of acceptability within a setting, user recruitment and engagement, and/or users' and providers' actual experiences with an intervention. Feasibility data was collected in four out of nine (44.4%) studies; however, two of these studies only referred to feasibility as part of an acceptability measure and did not report specific feasibility data (Schleider et al., 2020b; Shroff et al., 2023). The remaining two studies operationalised feasibility as referral and engagement rates (Moor et al., 2019), and clinician time spent per participant, incidents of therapist support above and beyond what was intended, and adverse events (Vigerland et al., 2024). Moor et al. (2019) found that referral rates increased throughout the monitored four-year implementation period, with a steady rate of monthly referrals observed from the first year onwards, and less than half of eligible referrals declined to engage. In Vigerland et al.'s (2024) study, the average therapist time for children and parents was 13.03 and 15.2 min, respectively, and less than half of users received additional support, including face-to-face sessions (Vigerland et al., 2024). Suicidal thoughts, school meetings, medication, etc., were the most common reasons for additional support (Vigerland et al., 2024). Overall, the findings of these studies indicated that primary care delivery of a DMHI is feasible.

### 3.9 Adoption

Adoption refers to the intent, initial decision, or action to try or deliver an intervention (Proctor et al., 2011). For example, whether people engage with an intervention may include the number of users who register or begin treatment, or the number of providers who refer an intervention. Adoption data was collected in all the included studies, although only eight out of the nine studies were explicit in their intent to collect adoption data. Eisen et al. (2013) was the only study that explicitly referred to adoption, and in their study, adoption data pertained to provider adoption (i.e., providers who participated in the implementation). All studies in this review provide information on the adoption of the intervention by reporting user statistics. For example, Vigerland et al. (2024) were not explicit about their intent to collect adoption data; however, their study provides information on the adoption of the intervention by reporting the number of users who were referred, registered, and engaged in treatment. Studies in this review referred to adoption as uptake, engagement, usage patterns, and referral patterns. Uptake, engagement, and usage patterns captured data on who adopted and began the program, whereas referral patterns measured provider adoption (i.e., which service providers were adopting the program by referring users).

#### 3.9.1 User adoption

The number of users tended to be higher among open-access and/or widely disseminated programs compared to isolated primary care settings. For example, March et al.'s (2018) large-scale dissemination of BRAVE Self-Help, with no referral or geographical requirements, reported 4425 users, whereas in the Moor et al. (2019) study, the same intervention delivered with minimal therapist assistance, and available only through professional referral and to people living in a particular geographical location, attracted only 1026 users. In both studies, the recruitment period extended beyond a year, and the intervention component differed only in terms of therapist support and referral source; yet uptake differed drastically. Conversely, one primary care-delivered intervention attracted over 5000 users (Alvarez-Jimenez et al., 2024); however, the intervention was widely disseminated and implemented across multiple clinics and locations. All but one of the open-access studies attracted over 1000 users (Schleider et al., 2020b). In a broader context, the findings further indicate that adoption is higher among non-ethnic groups (Eisen et al., 2013; Gee et al., 2024; Shroff et al., 2023).

#### 3.9.2 Provider adoption

Four of the nine studies (44%) collected data on provider adoption (Eisen et al., 2013; March et al., 2018, March et al., 2021; Moor et al., 2019), and all but one examined referral patterns. Referral patterns differed across the studies, but results show that school-based and external health professionals (i.e., family doctors, psychologists, or social workers) tended to be the highest form of referral. However, one study examining an intervention for school-aged children (March et al., 2021) found more than 30% of referrals had come from school-based professionals alone.

### 3.10 Acceptability

From an end-user perspective, acceptability refers to the extent to which an intervention is satisfactory to the consumer; it is distinct from service satisfaction (i.e., wait times, provider interaction) and relates only to the treatment aspect of an intervention (Proctor et al., 2011). For example, did users find the intervention useful and/or in line with their expectations of an agreeable experience (Hermes et al., 2019). Acceptability data was collected in five out of the nine (55.5%) studies and was most often captured by author-developed program feedback, satisfaction, and acceptability scales (Alvarez-Jimenez et al., 2024; March et al., 2018; Schleider et al., 2020b; Shroff et al., 2023). In contrast, Vigerland et al. (2024) recorded the proportion of people who accepted the offer to engage with the digital intervention as a measure of acceptability. Acceptability data was collected at different time points across studies and was most often collected from users who had completed the intervention (i.e., after completing all modules). Conversely, in the March et al. (2018) study, acceptability data was collected after sessions 4, 7, and 10 (final session). Given heterogeneity in acceptability measures, differential patterns of acceptability across self-guided and supported, depression and anxiety, and open-access and clinic-delivered interventions could not be made. Overall, the interventions in the included studies were found to be acceptable based on each study's requirement for acceptability endorsement.

### 3.11 Effectiveness

All studies identified in this review indicated that community-implemented DMHIs can be effective for lowering symptoms of anxiety and/or depression in children and adolescents. All but two studies reported effect sizes (Eisen et al., 2013; March et al., 2021), though the March study reported on the clinical significance of the improvements. Improvements in anxiety across studies showed small to large effects (Gee et al., 2024; March et al., 2018; Moor et al., 2019; Vigerland et al., 2024), while improvements in depression scores showed small to medium effects (Schleider et al., 2020b; Shroff et al., 2023). Alvarez-Jimenez et al. (2024) reported improvements in both anxiety and depression with small to medium effects. March et al. (2021) reported reductions in user anxiety and found that users with moderate anxiety severity obtained clinically meaningful benefit from the program.

Improvements in anxiety and/or depression across self-guided interventions (i.e., BRAVE Self-help, Project YES, Lumi Nova) showed small to medium effects. In contrast, therapist-assisted interventions showed small to large effects (BRAVE Therapist-assisted, BIP Anxiety, MOST). BRAVE Self-help (March et al., 2018) and BRAVE Therapist-assisted (Moor et al., 2019) both demonstrated medium effects in reducing anxiety. Interventions delivered in primary care settings produced small to large effects in reducing anxiety and/or depression (BRAVE Therapist-assisted; Lumi Nova; BIP Anxiety; MOST) while open-access interventions (BRAVE Self-Help; Project YES) demonstrated small to medium effects. Follow-up data was reported in one study and indicated continued reductions in anxiety (Vigerland et al., 2024).

### 3.12 Implementation strategies used

Only one study identified by this review explicitly stated the intent to use an implementation strategy to guide the implementation of the digital intervention; however, the details were not reported in the included study and were not yet available elsewhere (Alvarez-Jimenez et al., 2024). While the intent to use a strategy was not explicitly stated in the majority of the studies, all engaged in some form of dissemination effort; thus, an approach to dissemination could be inferred. The four open-access studies (March et al., 2018; March et al., 2021; Schleider et al., 2020b; Shroff et al., 2023) disseminated the program broadly to the community, with limited exclusion criteria, and relied on advertisement, word of mouth, and other health services (GP; online services, etc.) for recruitment to the program. Studies conducted in primary care settings (Alvarez-Jimenez et al., 2024; Eisen et al., 2013; Gee et al., 2024; Vigerland et al., 2024) identified people who presented to care and offered the digital treatment, while Moor et al. (2019) disseminated program information to health providers who referred eligible people.

Alvarez-Jimenez et al. (2024) and Eisen et al. (2013) were explicit in their use of the RE-AIM Framework (Glasgow et al., 1999) to evaluate the implementation of MOST and CATCH-IT in primary care settings. However, Alvarez-Jimenez et al. (2024) did not report the implementation outcomes in their study, and the Eisen et al. (2013) study did not provide a specific report on how the framework was used, instead reporting bespoke statistics to describe the implementation outcomes. Thus, none of the studies reported in this review conducted a clear evaluation of the effectiveness of implementation and implementation strategies.

## 4 Discussion

This review aimed to synthesise the literature on existing scale-up and dissemination efforts for child and adolescent DMHIs and determine the current state of implementation within the field. A systematic search and data collection guided by the PRISMA protocol found only nine studies that met the inclusion criteria. The purposeful use of implementation science was scarce, with only two studies explicitly stating the use of an implementation framework to evaluate the implementation effort. The included studies indicate an attempt to collect data pertinent to implementation evaluation and engage with real-world effectiveness and implementation research; there was a lack of evidence for research that planned and tested implementation strategies to disseminate child and adolescent DMHIs for widespread adoption. This review indicates evidence for the effectiveness of child and adolescent DMHIs when disseminated to community users outside of a tightly controlled research trial, however, effectiveness must be interpreted with caution due to a lack of follow-up data, small effect sizes, and in some studies a lack of reporting key information, such as the proportion of the sample who were within clinical ranges, and whether changes were clinically meaningful. This review found that a large proportion of community child and adolescent users engage with digital treatment; however, many who register interest do not begin, drop out after assessment measures, or fail to complete even one session. Yet, for those users who do engage, results indicate that young people and parents find DMHIs to be acceptable and beneficial.

### 4.1 Child and adolescent DMHIs

This review indicates that a range of child and adolescent DMHIs are being disseminated across different real-world settings. Models of therapy differed; however, CBT was the most commonly used model in the digital interventions. CBT has primarily been the model of choice for digital interventions, and CBT delivered via the internet is well known to produce results comparable to face-to-face CBT for child and adolescent depression and anxiety (Khanna and Kendall, 2010; Podina et al., 2016). Internet CBT (iCBT) may be the easiest model of care to disseminate at scale, given its ability to be easily delivered in an internet format and its capability to be self-directed or delivered with minimal assistance (Andersson et al., 2019; Lattie et al., 2022). Other therapeutic models, such as behavioural activation, are being used and presented as viable options for digital interventions. The majority of interventions in this review were delivered in a self-help format, which presents as a feasible option for large-scale dissemination because it removes the need for therapist integration, and access barriers. However digital interventions delivered with parental and/or minimal therapist support also present as achievable and feasible for community dissemination, although they typically require integration with professionals or services in some way. Despite some of the included interventions having no requirement for users to present with elevated symptoms, many of the interventions required a professional referral to access intervention content. This model of care requires the availability of professionals and the ability for young people to present to a service, both of which may present a major barrier to access (Gulliver et al., 2010; O'Brien et al., 2016). In this review, interventions that were open-access and did not require integration with a health service (i.e., BRAVE Self-Help & Project YES) presented a viable option for removing access barriers.

Many of the included interventions followed a similar format to that of standard face-to-face therapy; users engaged in a symptom screening process followed by the delivery of sequential, week-to-week sessions. Most interventions in this review provided treatment aligned with the symptoms targeted by the intervention (i.e., depression or anxiety) but did not personalise treatment modules to the individual's symptom profiles. However, one intervention did recommend the psychotherapeutic content to engage with, based on assessment responses (i.e., anxiety or depression content; Alvarez-Jimenez et al., 2024). Findings suggest that community-delivered digital interventions can serve as comprehensive models of care and be delivered in a manner similar to face-to-face therapy, even when in an open-access, self-directed format. However, a lack of tailored treatment may limit sustained engagement with digital interventions. In this review, it was observed that in most studies, less than half of the prescribed sessions were completed by users. Tailored iCBT programs may be one way to increase adherence by accounting for differing symptom profiles (Hadjistavropoulos et al., 2017; Johansson et al., 2012). The notion of treatment personalisation as a strategy for enhancing engagement has explicitly been endorsed by young people and parents engaging in self-help iCBT (Muller et al., 2022; Senyard et al., 2024).

### 4.2 Implementation science

This review indicates a lack of purposeful implementation research within the child and adolescent DMHI field. This implies that researchers have not yet fully transitioned from efficacy research to effectiveness research (i.e., implementation or process evaluations), where child and adolescent DMHIs are tested in and under real-world conditions. The included studies indicate methods and terminology that best relate to efficacy or effectiveness trials, rather than drawing from and being guided by implementation science. For example, studies focused heavily on determining whether the program was acceptable and effective for users, but rarely factored in methods to sustain uptake, adherence, and widespread adoption of the digital interventions in the relevant setting.

This review demonstrates that implementation outcomes are often operationalised and used in a way that aligns with the study's objectives, rather than implementation science frameworks. Proctor et al. (2011) determined that implementation outcomes had many definitions within the literature, though outcomes were often inferred or measured through attitudes, opinions, intentions, or behaviours. This was highlighted by the included studies in the current review, which often inferred implementation outcomes and/or measured the same aspect of implementation but referred to it differently across multiple studies. Similar to other implementation research, fidelity and acceptability were two of the most reported implementation outcomes in the included studies, while penetration, sustainability, and costs were collected less; and implementation strategies remain under-examined and -tested in real-world contexts (Wozney et al., 2018; Proctor et al., 2023). Researchers are now challenged with the successful implementation of interventions into the community, and research is required to develop implementation strategies for widespread dissemination of DMHIs. Without this information from a wide variety of interventions and settings, it is difficult to determine what works where and why.

The shortcoming of cost data in the literature for child and adolescent DMHIs has been criticised, while claims of digital interventions being associated with lower costs have remained unsubstantiated (Boydell et al., 2014; Hollis et al., 2017). The results of included studies unfortunately lend no further insight to the cost-effectiveness of DMHIs. Though, a recent review (Stanic et al., 2023) of economic evaluations of digital health interventions, including mental health, for child and adolescent populations, found that in 82% (18/22) of the included studies the digital intervention was more cost-effective or cost saving than with nondigital standard of care. Freely available health resources lend the biggest potential in reducing health disparities by providing access to users of all socioeconomic backgrounds and to those who are less served by traditional services such as those living in rural and remote locations (Muñoz, 2010). Cost evaluations need to be considered alongside efficacy and effectiveness evaluations given economic considerations in the implementation of interventions (Graeff-Martins et al., 2008; Ross et al., 2016). The dearth availability of sustainability and cost related data of child and adolescent DMHI's in the literature limit conclusions around the potential for DMHIs to improve health outcomes at low costs.

These findings may be attributed to the relatively recent emergence of literature on implementation strategies and methods for disseminating digital health interventions, including those focused on mental health (Graham et al., 2020; Hermes et al., 2019; Matson et al., 2025). Furthermore, the literature focuses heavily on strategies for implementing interventions into established healthcare settings, where service-level (i.e., professional or system) change is the primary focus of implementation efforts (Powell et al., 2012, Powell et al., 2015). However, open-access, self-directed interventions pose a practical option for increasing reach and scalability of psychological interventions (March et al., 2018; March et al., 2021; Muñoz et al., 2016). Yet, strategies for implementing widespread open-access interventions, particularly those targeting children and adolescents, remain underrepresented in the implementation literature. To combat the research-to-practice gap, hybrid effectiveness-implementation trial designs have been proposed (Curran et al., 2012; Matson et al., 2025). This method of research can accelerate the translation of interventions to practice by eliminating the need for separate effectiveness and implementation studies (Lane-Fall et al., 2019; Matson et al., 2025). Matson et al. (2025) propose a framework for developing hybrid effectiveness-implementation trials for digital interventions, specifically. While the framework proposed does not specify open-access programs, it provides a logical pathway towards more effective implementation research within the DMHI space. Digital interventions offer a potential solution to reducing treatment gaps in mental health; therefore, providing access to these interventions should be a primary goal for researchers. Engagement in implementation research, particularly through hybrid methods, may help achieve this goal.

### 4.3 Recommendations

Child and adolescent DMHIs are underrepresented within the implementation literature. Implementation research that plans, develops, and tests implementation strategies for community-delivered child and adolescent DMHIs needs to be at the forefront of the DMHI field. Research within the field needs to shift from efficacy trials to effectiveness-implementation trials, which test interventions in real-world contexts to identify strategies that encourage population-level adoption, implementation, and sustainability of child and adolescent digital models of care. Given that digital interventions exist in various forms, including prevention, single-session, education-based, and comprehensive models of care, it is imperative to determine which model of care works where and why, and how to best translate that model across different contexts. Therefore, recommendations include the need for more widespread and rigorous effectiveness-implementation research guided by implementation science, as well as a greater focus on developing a strategy for the widespread dissemination of open-access child and adolescent DMHIs. To achieve this, researchers need to engage with implementation science as a guide to their research at every step, from creation to implementation.

Acceptability was routinely collected by studies in this review, but like Wozney et al.'s (2018) review of child and adolescent eMental healthcare implementation research, satisfaction was the most common acceptability metric, and was collected using author-developed, self-report measures, predominantly post-treatment, therefore capturing only users who complete a certain number of sessions. The entire population of users should be considered to minimise overestimated satisfaction of DMHIs when delivered in the community (Wozney et al., 2018). By including users who fail to engage after referral, assessment, or one session, researchers are better equipped to understand why users choose not to engage or drop out following assessment measures or after a single session. This information is vital in creating high-impact interventions. This can be encouraged by providing an opportunity to engage with feedback surveys at any point of termination.

### 4.4 Limitations

The scarce availability of literature on the community implementation of child and adolescent DMHIs limited this review. Further, specific diagnostic criteria, namely the requirement for the included intervention to address anxiety and depression, limited the generalisability of these findings to broader mental health conditions like sleep or behavioural disorders. It is possible that digital interventions targeting sleep or behavioural problems may encounter different challenges and facilitators in implementation as they may be more caregiver-directed and will require examination to fully understand the implementation potential. Given the specific criteria surrounding implementation aims and metrics, conclusions about the real-world availability of digital interventions for child and adolescent users are limited to papers that addressed an aspect of implementation. It is possible that there may be other research that has indirectly reported on implementation outcomes that this review has missed through these criteria, however, this review highlights the need for more formal examinations of implementation outcomes. Additionally, only peer reviewed, English written research was considered in the search and inclusion criteria of this review, meaning any unpublished research in progress or non-English papers were not accounted for or included. Lastly, Heterogeneity of included studies and interventions prevented a meta-analysis, and in some cases limited identification of patterns and meaningful data comparison across studies.

## 5 Conclusions

Not only does community implementation research examine how DMHIs are received in the real world, but it also presents an opportunity to increase community access to evidence-based practices. This review indicates that implementation science within the DMHI field for child and adolescent mental health is lagging. Given the current state of mental health, the need for robust implementation research must be at the forefront of DMHI research. By engaging in real-world research, researchers may be better equipped to identify, develop, and test implementation strategies that enhance the success of DMHIs when deployed in the community. By engaging in effectiveness research that collects implementation data in real-world contexts, researchers can better understand their target audiences and are better equipped to increase the sustainability and dissemination of DMHIs for children and adolescents.

## Funding

This work was funded by the 10.13039/501100000925National Health and Medical Research Council — Medical Research Futures Fund [APP1179490]. This review was conducted as part of the first author's PhD project, for which the 10.13039/501100001795University of Southern Queensland has awarded a Research Training Program stipend.

## Declaration of competing interest

The authors declare the following financial interests/personal relationships which may be considered as potential competing interests: Sonja March reports financial support was provided by 10.13039/501100000925National Health and Medical Research Council (GNT 1179490). Author SM acknowledges that although intellectual property for BRAVE-ONLINE is owned by UniQuest/the University of Queensland, they may potentially benefit from royalties related to the program in the future. SM is also on the extended Editorial Board for Internet Interventions. If there are other authors, they declare that they have no known competing financial interests or personal relationships that could have appeared to influence the work reported in this paper.

## Figures and Tables

**Table:** 

Search category	Included search terms
Population	Child OR adolesc* OR teen OR youth
Mental health	Anxiety OR depression OR internalising
Intervention type	CBT OR treatment OR intervention OR program OR therap* OR “cognitive behavi*”
Intervention mode/delivery	Online OR internet OR web-based OR app OR digital OR mobile OR smartphone OR e-health OR eHealth OR mHealth
Implementation	Implement* OR dissemin* OR translat* OR deploy OR intergrat* OR adopt* OR “program evaluation” OR uptake OR acceptab* OR usage OR fidelity OR penetration OR sustainab* OR cost OR innovati* OR reach OR evaluat* OR feasibility OR effective*

*Note*. The search string was adapted to a simple search to meet Science Direct search criteria: (child OR adolescent AND anxiety OR depression AND program OR therapy AND online OR digital AND implementation).
